# A public health approach to palliative care in the response to drug resistant TB: an ethnographic study in Bengaluru, India

**DOI:** 10.1186/s12904-018-0374-5

**Published:** 2018-10-31

**Authors:** Joseph M. Sawyer, Rahul Asgr, Florence N. Todd Fordham, John D.H. Porter

**Affiliations:** 1 Marie Curie Palliative Care Research Department, UCL, 6th Floor, Wing B, Maple House, 149 Tottenham Court Road, London, W1T 7NF UK; 2 Society for Community Health Awareness Research and Action, Public Health, Bengaluru, Karnataka India; 3 Departments of Clinical Research and Global Health and Development, London School of Hygiene and Tropical Medicine, Keppel Street, London, WC1E 7HT UK

**Keywords:** TB, MDR-TB, Palliative care, Suffering, Drug resistance

## Abstract

**Background:**

The treatment of Multidrug-Resistant Tuberculosis represents one of the most significant challenges to global health. Despite guidance on improving treatment outcomes, there is little focus on how to support individuals in their suffering. Palliative care is therefore proposed as a necessary component in the global strategy to fight Tuberculosis. We aim to describe the informal resources and networks available to persons affected by Multidrug-Resistant Tuberculosis, how they are accessed and how they are integrated into everyday lives.

**Methods:**

In-depth ethnographic research was conducted in Bengaluru, India. Informal interactions and observations were recorded across a range of palliative care and tuberculosis treatment providers over a month-long period. In addition, ten individuals with Multidrug-Resistant Tuberculosis were asked for in-depth interviews, and five agreed.

**Results:**

Multidrug-Resistant Tuberculosis caused a dynamic chain of events that transgress through physical and psychological domains to cause human suffering. Participants utilised support from their family and friends to build a network of care that was of therapeutic benefit. Informal care networks were similar to the holistic model of care practice by specialist palliative care services and represent an underused resource with enormous potential.

**Conclusion:**

Patient suffering is poorly addressed in current Tuberculosis treatment programmes. A community-based palliative care approach may extend peoples’ support networks, helping to alleviate suffering. Further research on existing support structures and integration of these services into Tuberculosis control programmes is required.

## Background

Tuberculosis (TB) is amongst the top 10 causes of death worldwide and in 2017 it caused an estimated 1.3 million deaths, the highest from any single infectious agent [1]. Attempts to control the disease reflect both advancements in medicine but also how society cares for its most vulnerable. Indeed, the UK achieved a steep decline in the burden of TB before effective chemotherapy became available, indicating the importance of socio-economic factors in disease control [2]. A dominant pharmacological approach has seen these matters take a back seat whilst problems relating to access and adherence to treatment have grown [3]. The world is now facing TB in its resistant form, with the situation described as a global public health crisis [1].

Multidrug-resistant-TB (MDR-TB), defined as resistance to at least isoniazid and rifampicin, is a life-threatening condition posing an international threat to public health. In 2017, the WHO estimated 558,000 incident cases of Rifampicin resistant (RR) TB worldwide, of these an estimated 82% had MDR-TB, resulting in 230,000 deaths [1]. MDR-TB has a global treatment success rate of 55%, whilst extensively drug resistant TB (XDR-TB), a subset of MDR-TB with added resistance to fluoroquinolones and second line injectable drugs, is at 34% [1]. These statistics only partially tell the story as many people are left undiagnosed, are lost to follow up or do not have their treatment outcomes evaluated.

India has the largest burden of TB related disease however the true effect of M/XDR-TB is difficult to quantify in part due to a lack of specialist drug sensitivity testing. Of the 558,000 MDR-TB cases globally almost half (47%) were in India, China and the Russian federation [1]. In 2016 there was an estimated incidence of 147,000 cases of RR/MDR-TB in India, with a treatment success rate of 46% for MDR-TB and 29% for XDR-TB [4]. As such the country is a major focus for TB prevention and control programmes.

As we face the challenge of finding new drugs and improving diagnostics, we must not neglect the care needs of those suffering from potentially terminal disease. This is acknowledged in the WHOs End TB strategy which outlines a vision of zero deaths, disease and suffering due to TB [5]. This bold vision requires innovative approaches that work in conjunction with communities whilst promoting human rights, ethics and equity [6]. With this in mind, the WHO have advocated for palliative care in the global response to MDR-TB [7–9]. Palliative care provides a holistic network of support with a focus on human suffering that spans physical, psychological, social and spiritual needs [10]. Fundamental to the specialty is the concept of ‘total pain’ which takes into consideration not just physical distress but also mental, emotional and social pain [11].

For a long time, palliative care has been associated with care of the terminally ill towards the end-of-life. However, there is a growing recognition that its services can have profound impact when integrated alongside curative treatments in non-terminal conditions [12]. Many larger hospice organisations now offer palliative care for serious, non-terminal conditions for the purpose of improving quality of life and aiding the recovery process for both the individual and their family. With the evolution of cancer treatments many malignant diseases are now curable yet the need for palliative care services continues to grow. The focus of care has shifted from purely end-of-life care towards complex symptom management and emotional support in order to support people through the ordeals of chemotherapy and into the survivorship phase where people are often left with untreated chronic symptoms that have a dramatic impact on health. There are direct parallels with this and the treatment of tuberculosis and as such the role of palliative care warrants further evaluation.

Specialist palliative care has been present in India since the 1990s although coverage remains poor [13]. This is a problem facing palliative care globally; in response, a public health approach has been proposed.

First outlined by Kellehear, the movement is characterised by social efforts designed to build cohesive community networks that provide person centred holistic care [14] [15]. These networks are often described as ‘compassionate communities’ [16–18]. Networks may either be formal, composed of professionally facilitated links within a healthcare setting or informal, compromising an individual’s personal social networks and support structures built up over time. Under this model, issues such as those seen in TB; loneliness, stigma and inequitable care access, are community issues best tackled by its constituent members rather than healthcare professionals alone.

This approach has led to a wave of community-motivated interventions. From the mobilisation of resources through naturally-existing or externally-facilitated social networks, to the direct support and training of volunteers, there is now an international movement to promote health at the end-of-life [19]. This work is potentially transferable to MDR-TB and could address issues around treatment adherence, infection control at home, nutritional support and treatment withdrawal.

The concept of ‘compassionate communities’ in India is relatively new, however developments have occurred In Kerala, where there is a community-led initiative aiming to provide home-based palliative care [20–22]. How this model is integrated across a culturally diverse nation with the added influence of traditional healing systems has not been explored.

### Aims and objectives

We explore the context of disease and disease management from the individual’s perspective. Using this as a backdrop, we aim to describe the informal resources and networks available to people with MDR-TB, how they are accessed and integrated into everyday lives at a cross-section in time. Framing this in the context of a public health approach to palliative care, we hope to provide evidence for the development of a community-led supportive care programme specific to MDR-TB.

## Methods

### Conceptual framework and literature review

**Fig. 1:**
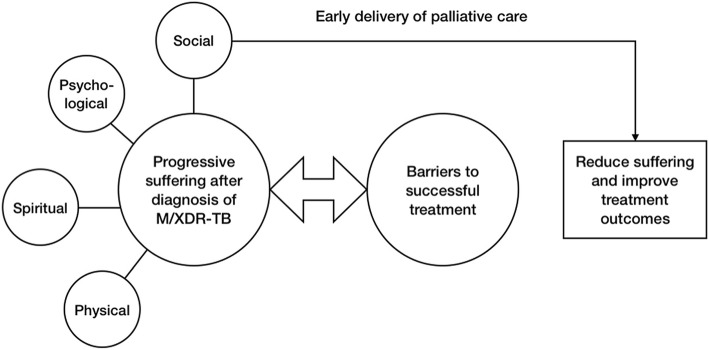
Conceptual framework

A conceptual framework was created to guide the study and data analysis (see Fig. 1). This was based upon the initial literature review, which demonstrated the structural components of a palliative care programme and how these may accommodate the different modalities of suffering experienced. The following search terms were used across Medline and Embase; exp. Tuberculosis, Multidrug-resistant/ or exp. Extensively drug resistant Tuberculosis/ or exp. Tuberculosis/ or exp. Mycobacterium tuberculosis AND Palliative Care OR palliat* care or palliat* medicine or hospice or Psychol* support or spirit* support or Soc* support. After eliminating duplicates and reviewing documents available in full text, a total of 57 relevant articles were identified. The search was updated in 2018 with the same terms and revealed a total of 61 relevant articles.

### Study setting

The study was conducted in Bengaluru (Bangalore), India where urbanisation has brought an influx of migrants from neighbouring states resulting in rich linguistic and cultural diversity. Healthcare is predominantly provided by a westernised privately funded system. However, Bengaluru is also home to AYUSH (Department of Ayuverda, Yoga and Naturopathy, Unani, Siddha and Homeopathy). These traditional Indian healing systems offer a holistic approach to disease prevention and cure and have a significant but poorly quantified impact on the delivery of healthcare in India [23]. Formal networks of care were provided through institutions of healthcare including those private and state funded. Informal networks of care were more subtle and ranged from family and community support to the care received from religious institutions and the local environment.

### Study design

This study used qualitative ethnographic methods. In-depth interviews and informal observations formed the main data collection methods.

### Interviews

Interviewees were selected by convenience sampling. They were recruited from the respiratory clinic attached to St Johns Medical Centre (SJMC), Bengalaru, India. Telephone contact was made by the lead nurse who was familiar with all participants. A preliminary meeting was arranged to introduce the research team and discuss consent prior to interview (see Table 1 for research team).

**Table 1:** Research team members and their background

Team member	Role	Background/qualifications
Researcher 1	Lead researcher and instigator of conceptual design. Author of field notes and observational study. Present during interview and recruitment process, contributed probing questions via interpreter. Lead for data analysis and coding. Introduced to participants as the lead for research alongside information sheet.	Native to UK and foreign to the studied environment. UK based general medical training and fully qualified clinician at the time of study. Basic training in social science methodology.
Researcher 2	Translator for interview and recruitment process. Helped conduct interviews using topic guide. Provided valuable reflections and insights that contributed to data analysis.	Native to India. Fluent in all local dialects. Indian based medical training and fully qualified clinician. Basic social science training and public health training.
Researcher 3	Supervisory role, contributed to theoretical development of the study and data analysis through discussion of data and evaluation of themes.	Native to UK. Medical training in the UK, public health training in the USA and UK. Extensive experience in public ethics of TB treatment. Knowledge and experience of AYUSH.

Interviews were conducted in the summer of 2014 with the use of a topic guide created following a literature review. A pilot interview was held on arrival to the study site and the topic guide refined. Interviews were conducted by team members 1 and 2. Interviews were conducted in the participants’ preferred language: Kannada, English, Tamil or Hindi. Where the interviews were not conducted in English, translations were verbatim and conducted in real-time. Interviews occurred in clinics or participant’s homes and lasted up to an hour. Interviews were audio-recorded and transcribed in English; emerging themes were recorded in a contact summary form on the day of interview. The audio-recording and written translations were subsequently reviewed by the original translator and discrepancies corrected. Reflexivity is key when conducting ethnographic methods, and thus the social background and personal assumptions of the translator were considered when reviewing transcripts.

Ten participants were approached to take part. One withdrew after interview, four declined to take part and one participant did not have capacity to consent. The remaining participants are summarised in Table 2. The interview with participant four was done in conjunction with his wife (participant 5). A significant theme that hindered recruitment centred on the process of consent and the various cultural forces that impact on this process.

**Table 2:** Patient demographics and Interview Code: MP = Male Participant, FP = Female Participant, FPFM Female participant, Family member

Characteristics	Participant 1	Participant 2	Participant 3	Participant 4	Participant 5
Age (years)	50–60	20–30	20–30	40–50	40–50
Sex	Male	Female	Female	Male	Female
Treatment stage	Completed	Completed	Continuous phase	Continuous Phase	N/A
Previously treated for TB	Yes	Yes	Yes	Yes	N/A
Socio-economic status	Low	Middle	Middle	Upper	Upper
Living arrangement	Joint family	Nuclear family	Nuclear family	Nuclear family	Nuclear family
Marital status	Married	Single	Single	Married	Married
Language spoken	Kanada	English/Kanada	Tamil	Hindi/English	Hindi/English
Occupation	Labourer	Student	Student	Office worker (IT)	Housewife
Religion	Hindu	Christian	Atheist	Christian	Christian
Educational status	No formal education	Formally educated	Formally educated	Formally educated	Formally educated
Interview code	MP1	FP2	FP3	MP4	FPFM5

The principle of autonomy, as understood by the Western bioethics, forms the cornerstone to informed consent. In the Indian context, paternalism and patriarchy may influence decision making. Whilst this is perhaps more common in the rural setting, Bengaluru has a diverse population representing great breadth in cultural norms and this issue was frequently encountered. In addition, many participants who were suffering with advanced and disabling disease were too fatigued and fearful to take part. One participant in particular, who was from a low socio-economic background, became concerned that involvement in the study would impact negatively on his family, disrupt his treatment options and the relationship with his treating medical team.

***“I have met with X twice to discuss the study, each time there appear to be new concerns. Today X was fearful of having his treatment withdrawn as a consequence of speaking to me…not even the local DOTS (Directly Observed Treatment, Short-course) provider [whom X was friendly with] could pacify X’s concerns….”*** *Excerpt from field diary: 5th July 2014.*

Ethical issues surrounding the consent process were discussed with the wider supervisory team who provided valuable insights into issues around cross-cultural social dynamics as well as research and trust ethics. These discussions contributed to the analytic phase.

### Informal observations

Informal observations were conducted across the sites mentioned in Table 3. Healthcare professionals were shadowed in their work and informal conversations were held when opportunity arose. Data was collected in the form of field notes. Informal observation facilitates a deep review of the social world in which the study is situated. However, this method also raises issues of subjectivity and researcher bias. This is pertinent as the study was conducted in Bengaluru, where language barriers and cultural considerations positioned the researcher in a new social context. Therefore, it is important to reflect on the research process, the challenges of reflexivity and the influence of researcher perceptions.

**Table 3:** Description of study sites

Site	Description
Site 1	A large Catholic funded private hospital practicing western medicine. Discussions were held informally with doctors and observations of MDTs, the ward environment and outpatient services were conducted.
Site 2	An autonomous institute specialising in western medical treatments of respiratory disease. Informal observations were held during a visit designed for recruitment purposes.
Site 3	An interdisciplinary resource group of community health professionals utilising multiple pathways to facilitate and promote the goal of Health for All. It focuses on public health system development, action on the social determinants of health and community action for health with a social justice perspective. Constituent members had a range of expertise spanning the social sciences, allopathic medicine, traditional holistic Indian healing systems (AYUSH). Observations were conducted over a month long period and informal conversation held with constituent members on issues ranging from the impact of AYUSH, the relevance of palliative care to TB and the impact of trust ethics on recruitment to the study. This helped enable me as the primary researcher to become more embedded in the culture and traditional practice.
Site 4	A traditional hospice based on the UK model. Practice included western medicine, social work and chaplaincy and spiritual support. Informal conversations were held with members of each of these disciplines during a day visit.
Site 5	A traditional hospice based on the UK model. Conversations were held with nursing staff and observations conducted during a day visit.
Community health centres	Patients were met and consented for recruitment at various community health centres scattered across the city. The process of consent would often take several meetings during which observations were conducted.
Churches	Local Churches of catholic denomination were visited and observations held. The role of spirituality was discussed informally with the community of brothers.
Temples	Small temples were scattered throughout the city and visited for the purposes of observation only.
Community settings	On several occasions people were visited at home to discuss enrolment in the study. Observations of their living environment and surrounding area were conducted.

Haraway states that self-reflexivity is defined by an “introspective stance” towards one’s own conception of information, provoking an awareness of a researcher’s positionality [24]. With respect to this study the lead researcher was foreign to the studied environment. The work also required a transition from a western biomedical approach towards more holistic constructs. In order to achieve this, a period of two weeks was spent meeting members of SOCHARA (Society for Community Health Awareness Research and Action) and learning about traditional Indian healing practices. SOCHARA is an innovative community of professionals and activists who facilitate community health and well-being using an equity, rights and health determinants perspective. Spending time here allowed the principal researcher to become aware of themes that arose in forthcoming data collection.

To further counter the effects of positionality, and the potential influence on the construction of knowledge, a field diary was kept to critically and constantly analyse researcher perceptions. This reflexive fieldwork practice prompted a more conscious encounter with the multi-faceted aspects of the study site, whilst still acknowledging the situatedness of the researcher, giving rich insights into the doctor-patient relationship in the context of overarching professional structures of care in Bengaluru.

### Data analysis

To analyse the ethnographic field notes, a traditional approach of immersion in the text through repeated reading was used. Interviews were analysed via a process of coding based on recurring meanings and ideas [25]. NVivo was used to support a preliminary framework analysis to gain familiarity with the data before developing more detailed thematic analysis.

During thematic analysis coding groups were built up in an iterative process across the whole dataset. This allowed integration between the field notes and interview data. Recurring ideas were grouped together into higher-level themes [25].

#### Ethical considerations

The study was approved locally by the St Johns Medical Centre ethics committee of Bengaluru, India (ref 78/2014, June 2014) and by the London School of Hygiene & Tropical Medicine Research Ethics Committee (ref 7617, June 2014). Each participant was asked to read an information sheet and given opportunity to ask questions before giving written consent to take part in the study. Where participants could not read, this was read to them in their preferred language. All participants have given written consent for the publication of anonymised results.

## Results

The findings are discussed according to the themes identified. The first section explores the human context in which biomedical disease management occurred. The second section explores the informal networks people established and how they were integrated into everyday life.

### Human context of disease

#### Realms of suffering

The medical management of MDR-TB was found to occur in the context of profound human suffering. This theme emerged through discussion and observation.

***“Physically X looks very weak and X must be very frightened”*** *Excerpt from field diary: 5th July 2014.*

Interview data revealed suffering to occur according to a disturbance in human connection, either with the self, with others or within a spiritual network.

Inevitable thoughts on death created an uneasy relationship with ‘the self’ as participants began to examine their existence in the context of MDR-TB. This ranged from inner turmoil to thoughts of suicide.

***“I didn’t know what else to do so I thought killing myself was the best thing”*** *MP1.*

This led to a breakdown in relationships within the family or wider social network.

***“...because he can’t share his stress with some other [person], he is speaking [only] with me, maybe fighting with me”*** *FPFM5.*

In search of stability people sought solace in religion, whereas others found this to be another source of uncertainty and grief.

***“So when I was not able to get up my sister took me to the church in Madivalla, they said a prayer and I was able to stand”*** *MP1.*

***“He lost a little trust in God…faith was reduced”*** *FPFM5.*

This lead to the understanding that spiritual suffering was not restricted to religion, instead representing an unmet human need that encompasses and transcends the traditional bio-psycho-social model. Imbalance within someone’s spiritual network was a principal theme within the data and represented a disturbance in the connection people felt with themselves, others around them, and their living environment.

***“…my elder sister had two children. I missed to kiss them and hug them, really I missed it. I keep wanting to kiss and hug them but I am worried [about disease transmission], I maintain the distance, I do not kiss her, I do not share food or anything*** *…” FP2.*

#### Points of suffering

The relationship of human suffering to the context of biomedical treatment models highlighted three themes upon which suffering was noticeably triggered or accentuated; prognostic uncertainty, vulnerability in disease disclosure and the trauma of drug treatment.

Prognostic uncertainty caused spiralling doubt that would permeate through anticipated major life events. All patients had been told their disease was ‘definitely’ curable however limited information had been given beyond this.

***“...no council was done they just made a diagnosis…” FPFM05.***

This fed into the aura of uncertainty that would lie on the path ahead, creating anxiety and apprehension.

***“I don’t know, I believe that it will be curable, I have been told that there is a 100% cure, I have been taking the treatment so I believe that it will be cured, I really don’t know, you should ask the doctor or the sister about this...”*** *FP03.*

***“…it is common for respondents to convey a sense of positivity with respect to their prognosis, however when given the opportunity to ask questions to me everyone wants to know why it [MDR-TB] has affected them, the likelihood of it coming back and the chance of cure…”*** *Excerpt from field diary: 5th July 2014.*

This theme later developed across the whole data set:

***“I keep on thinking whether it is curable or not. Then marriage will come and I am worried about it…”*** *FP2.*

In disclosing their diagnosis, participants faced a range of reactions that varied according to social context. Themes included disdain, pity, fear and aggression.

***“So they scold me because they know that I am having the disease, but what can I do? I just move away if they tell me to go, I cannot do anything about it…”*** *MP1.*

Vulnerability was also detected at the doctor-patient interface. In the context of privately-funded healthcare, participants were often subject to long in-patient admissions. This often represented a lack of participant involvement in decision making which came at a physical, psychological and financial cost.

***“Actually, no, hospital is not that clever, because they will think maybe it’s something else [not TB] and they will ask him to stay [in the hospital]…That made him feel even more stressed…”*** *FPFM5.*

Whilst TB chemotherapy was seen as the cure, it was in itself a source of suffering. Side effects range from disturbance in vision to lethargy, nausea and vomiting. In some cases, the cure became as much of a burden as the disease. One interviewee highlighted that as a parent, watching your child endure this suffering could lead to a vacillating appreciation of what may be right or wrong.

***“She [participant’s mother] kept telling me don’t worry. She is also crying, when they put [administer] the injections I will be crying and my mum also is crying”*** *FP2.*

### Resources, practices and support networks

Recognition of the importance of holistic care, and the role of the community in providing it, arose through observational data collected during a SOCHARA workshop:

***“…I am fascinated to learn of the concept of community health and it strikes me as being incredibly relevant yet almost completely unspoken of in the [UK based] National Health Service. The work done in communities here implies that medical services are not just a model or structure for consumerist use but embody a fluid process that enables/empowers people for collective action and care. Here the community is able to maintain health and provide health within its own means...this is a radically different approach that is well characterised by their ‘paradigm shift’ i.e. moving away from professional ‘control’, moving from a medical model to a social model, from patients to the individual, disease to health, providing to enabling, drugs and technology to knowledge and process...”*** *Excerpt from field diary 16th June 2014.*

Interview data developed this theme, highlighting participants reliance on their family and friends to provide supportive care. This ranged from practical help, such as transport and food, to a deeper level of care that embodied themes of compassion and love. This was demonstrated by one participant who could not cope with the sight of her tablets. Her father collected her tablets from hospital and her mother prepared them daily in a specially made box.

***“Yeah, she [Participant’s mother] only cuts my tablets and gives it to me. I did not see anything...So my mum gave me separate in the box also. That was the main thing; yeah I didn’t see a bunch of tablets…”*** *FP2.*

The assiduous nature of a mother’s care, combined with the sense of normality conveyed by family-led care, was vital to the participant’s well-being and ability to continue treatment.

In search of support of this kind, participants would explore human connections - both physical and metaphysical. This was demonstrated by a participant explaining how he survived feelings of self-harm and suicide:

***“So going to the church helped reduce that thought of killing myself, I was also admitted for two/three months in the hospital and this reduced that thought also…When I was in the hospital the doctors used to come and take care of me, give me tablets, the food was also free, I used to not think so much about the house and also there used to be five or six other patients in the ward and I used to talk to them and my time would pass so all these thoughts [of suicide] would not come”*** *MP1.*

From this, it became apparent that support structures were interdependent. This represented holistic care and, as a concept, was fundamental in addressing a state of equilibrium that we may call ‘health’. The holistic care observed was similar in construct and theory to that provided by a specialist palliative care service.

***“Because of all the support I am well now, without the support it would have been difficult”*** *MP1.*

In contrast to observed community support networks, medical structures of care promoted fear amongst the community, whilst demonstrating coercion as a means of control.

***“…sometimes when I didn’t come to the hospital the doctor used to come and give me the tablets, so if the doctor used to come home with the tablets the neighbours used to scold me saying that you have this disease, so from that day onwards I always used to come to the hospital and take the tablets myself.”*** *MP1.*

Importantly, community-led care occurred naturally and was initiated and provided for by lay people. In the hospice environment, the intervention was creating an environment where community support could flourish, thereby promoting and strengthening social networks. Observational data from hospices highlighted how the environment held and supported staff and patients alike.

**“** ***…I was struck by the importance of the environment in creating a positive and healthy atmosphere. This was mirrored by the staff who were engaged and enthusiastic about their work…it became clear that the medical consultation was only one step in the chain of support that assisted patients and their families back into the community or in the case of those with end stage disease, through the process of death….”***

***“After sharing lunch with the staff, we walked back through the grounds where X mentioned how they had built the surrounding gardens from scratch, he spoke of the positive atmosphere this created for him which resonated with my initial feelings on arrival.”*** *Excerpts from field diary 16th June 2014.*

These findings were in contrast to experiences from hospitals which were often overcrowded and fearful places.

***“I waited to speak to the Doctor for an hour and half, during this time I was surrounded by TB patients...Many were emaciated, some barely conscious...this felt like an isolating and fearful place full of pain.”*** *Excerpt from field diary: 4* ^*th*^ *July 2014.*

***“...actually they asked him to stay [in the hospital]...the environment he didn’t like because all the other patients are more severe [severely unwell], he was just started [on treatment]...he got even more scared around them...”*** *FPFM5.*

It was found that structures valuing human connections as therapeutic were of fundamental importance. Peoples’ individual stories, their resources, practices and networks of care, were brought together and integrated into everyday life through the creation of a social platform, be that a hospital ward, a park, a place of worship or a cinema.

***“I speak with my friends also like going to movies and things like that.”*** *FP2.*

***“I like to go to temples, I feel comfortable, I express my feelings with God…”*** *FP2.*

***“So I used to go and sit and parks, also I used to go and walk around”*** *MP1.*

Being able to ‘connect’ in the broadest sense of the word allowed for the development of a network that encouraged self-care and community-led care.

## Discussion

### Main findings

This study describes informal networks of care that are responsive and reflexive to the multi-dimensional human suffering caused by MDR-TB. Community based care networks were found to be theoretically similar to the model of holistic care enacted by specialist palliative care services. Participants built supportive networks through existing social platforms that intervened at focal points of accentuated suffering. This network appeared to work independently and sometimes at odds with a medical management programme that focussed on technology, drug treatments and specialist biomedical knowledge.

**Fig. 2:**
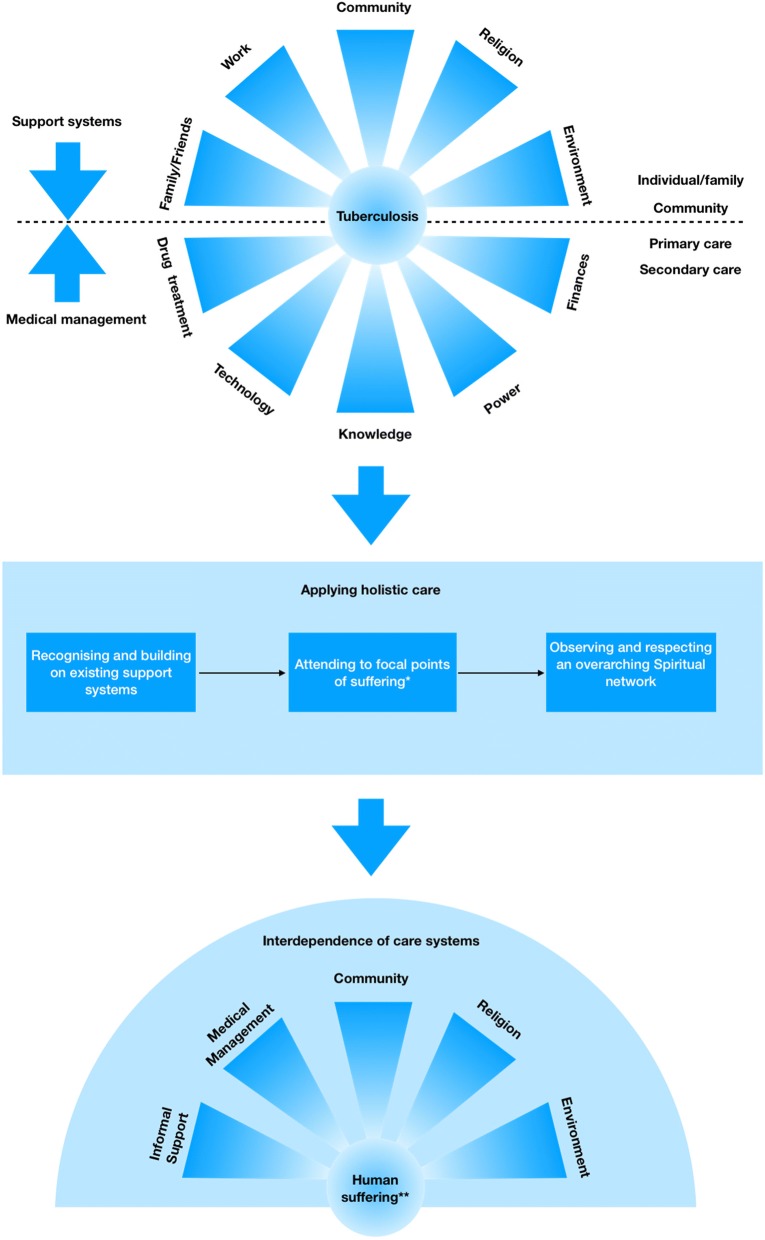
Schematic representation of themes and subthemes. *prognostic uncertainty, vulnerability in disease disclosure and the trauma of drug treatment. ** a disturbance in human connection, either with the self, with others or within an overarching spiritual network

These findings suggest that in order to achieve the vision for ‘zero suffering’ outlined as part of the WHOs End TB strategy there must be a robust capacity for delivering large scale, context specific and community centred palliative care services. Applying the principles of a public health approach to holistic palliative care allows human suffering to become the central focus as opposed to the disease. What follows is a recognition of the interdependence between supportive structures allowing them to realign and work in unison to help bring about meaningful outcomes at both the individual and population level (See Fig. 2 for schematic representation).

### Strengths and limitations

The rising calls for TB treatment programmes to integrate palliative care into their services have been met with a growing evidence base [26] [27]. Currently this focusses on symptom burden giving strong evidence for the integration of specialist palliative care alongside potentially curative options [26]. By describing human suffering in the context of MDR-TB we found suffering was poorly acknowledged by professional structures of care thus giving weight to this argument. In addition, we propose three focal points to suffering upon which interventions could be designed. These relate to a disturbance in human connection, either with the self, with others or within a greater, all-encompassing, spiritual network. The overarching domain of spiritual care represented the bond between individuals, families, communities and their natural environment whilst encompassing themes of love and compassion. This research was embedded in a culture of traditional holistic networks of care and is therefore culture and context specific.

Interviews were conducted and analysed with an explicit interest into how social networks and community structures related to the suffering experienced. Participants were found to depend on a network of family, friends and community members. Rather than compromising care, this was found to add quality and enhance relationships whilst allowing people to overcome physical and psychological barriers to treatment. This was in part due to care being individualised and person specific. This is congruent to the theories proposed by Kellehear in the development of ‘compassionate communities’ [18]. Most existing research is framed within advanced or incurable disease. Our results suggest there is scope to integrate this approach alongside curative interventions in order to tackle issues such as access and adherence to medications.

Traditionally, palliative care has utilised professional knowledge and skills to deliver such a service. However, in the context of limited resources, our study suggests that non-specialist measures from friends and family were used to good effect. There is an argument that the standard of care delivered by an attentive family, supplemented with access to relevant knowledge and information, would provide a standard of care greater than that achieved by professionals alone*.*

It is important to acknowledge the inherent methodological limitations to the study. As a non-native researcher entering an intensely emotional and culturally foreign context, researcher bias is an issue. As such, reflexivity during field work and during the evaluative process was of vital importance. Cross-cultural group discussion and cultural integration allowed the principal researcher to reflect upon his personal beliefs, values and professional identity and how they influenced the research process. It is also recognised that some themes will have been left uncovered in the study. With such cultural breadth in Bengaluru it was not possible to uncover the complexities in each unique social network with 5 participants from predominantly middle and higher socio-economic backgrounds. This combined with the highly personal and emotionally challenging nature of the interviews and the inherent language barriers, meant that some issues may have been left uncovered. Using the same translator who was native to the culture and briefed in the study objectives helped limit this impact.

### What this study adds

Our research adds weight to the call for integration of palliative care services into MDR-TB treatment programmes. By demonstrating the importance of community networks, and evaluating how they are accessed, we suggest a potential mechanism by which a sustainable palliative care service may be integrated into this specific setting. A public health approach to palliative care is growing a significant evidence base and palliative care as a speciality is ideally placed to drive forward such interventions [19]. Further research is required to understand the impact of social networks in this particular disease context across a range of settings.

## Conclusion

The findings from this study suggest that under the WHO’s End TB Strategy there is scope to integrate palliative care practice at local, national and international levels [9]. If we are to acknowledge the report’s ambition to reach zero deaths, disease and suffering whilst addressing the complex ethical issues surrounding treatment there is a desperate need for community empowered palliative care services to support on the issues raised throughout this paper.

Further research into existing support networks for both patients and carers is required in order to build a robust capacity for delivering palliative care in MDR-TB. To further understand this, longitudinal studies looking at how and why social networks evolve and adapt around an individual with tuberculosis over the course of treatment and beyond are essential. This must run in conjunction with attempts to influence society’s perceptions and reactions to death and dying. By recognising and empowering the work done by communities we can build an economically sustainable model of palliative care with a level of quality and continuity that is in tune with peoples cultural and social needs.

## Abbreviations

AYUSH: Ayuverda, Yoga and Naturopathy, Unani, Siddha and Homeopathy
MDR-TB: Multi-drug resistant TB
RR-TB: Rifampicin resistant TB
SJMC: St. Johns Medical Centre
SOCHARA: Society for Community Health Awareness Research and Action
TB: Tuberculosis
WHO: World Health Organisation
XDR-TB: extensively drug resistant TB
